# Delegates to the State Medical Association

**Published:** 1898-02

**Authors:** 


### Delegates to the State Medical Association.

Rusk, Texas, January 31, 1898.

*To the Physicians and Medical Associations of Texas:*

We, your committee, appointed in mass meeting assembled at Dallas, Texas, December 13, 1897, having assumed the arduous duties imposed, earnestly desire that each Medical Association in the State appoint delegates to meet us at the State Medical Association, and all physicians in counties where no Associations exist are requested to be represented, notwithstanding we will from time to time address you with circular letters, but there are matters of graver importance which demand a personal interview, and with the hearty co-operation of the physicians of Texas victory will crown our efforts. See circular letter No. 1, elsewhere. Fraternally,

H. O. Lyford, Chairman,
E. E. Gunn, Vice-Chairman,
J. G. Wiggins, Sec'y. and Treas.,
State Central Committee.

Office of Secretary, Rusk, Tex., Feb. 1, 1898.
Official Circular No. 1.

*Dear Doctor:*

As you are doubtless aware, we have been appointed a committee to formulate and prosecute a campaign in favor of the repeal by the next legislature of the obnoxious and iniquitous occupation tax unjustly imposed upon the members of our profession, and, as well, in favor of a measure to be presented to and passed by the 26th legislature protecting the public against medical quacks and humbugs. We have been much encouraged in our labors, but our pressing need is funds to pnt upon foot and carry to completion the work assigned. If you are in harmony with our purposes please remit us forthwith such sums as you may feel disposed to contribute to this cause, bearing in mind the expense of such a campaign is enormous, but a few dollars from each will carry the work to a successful termination, without which we cannot hope for victory. We cite you to the fact that the meeting of the physicians of this State convened and held in the city of Dallas on the 13th day of December, 1897, designated the undersigned as a committee to peform this labor. We hope to effect an active, vigilant organization in every county in the State, and funds are required for this work. Our labors are onerous, but we enter into the performance of our task with pleasure and pride, and pray your hearty co-operation. You will please recommend some active physician in harmony with this measure to be appointed chairman of a committee in your county, to assist us with the work. This is very essential, that we make the proper appointments.

Yours for the work,

Dr. H. O. Lyford, Chairman,
Dr. E. E. Guinn, Vice-Chairman,
Dr. J. T. Wiggins, Sec'y and Treasurer,
State Central Committee.

P. S.—Please address the Secretary, at Rusk, Texas, at once.

